# Supplementary material for: RT-qPCR Expression Profiles of Selected Oncogenic and Oncosuppressor miRNAs in Formalin-Fixed, Paraffin-Embedded Canine Mammary Tumors
Source: Animals (Basel). 2022 Oct 22;12(21):2898. doi: 10.3390/ani12212898 (PMC9654908; doi:10.3390/ani12212898)
Supplement: Supplementary file 1 [file animals-12-02898-s001.zip › animals-1957412-supplementary.pdf]

Table S1. List of predicted target genes for dysregulated miRNAs generated using the miRDB online analysis platform.

Target Rank	Target Score	miRNA Name	Gene Symbol	Gene Description
1	99	cfa-miR-18b	ESR1	estrogen receptor 1
2	99	cfa-miR-18b	PATJ	PATJ, crumbs cell polarity complex component
3	98	cfa-miR-18b	RORA	RAR related orphan receptor A
4	97	cfa-miR-18b	MAP7D1	MAP7 domain containing 1
5	97	cfa-miR-18b	BBX	BBX, HMG-box containing
6	96	cfa-miR-18b	MNDA	myeloid cell nuclear differentiation antigen
7	95	cfa-miR-18b	CREBL2	cAMP responsive element binding protein like 2
8	94	cfa-miR-18b	ERI1	exoribonuclease 1
9	94	cfa-miR-18b	ESF1	ESF1 nucleolar pre-rRNA processing protein homolog
10	93	cfa-miR-18b	TRAPPC8	trafficking protein particle complex 8
11	93	cfa-miR-18b	IRF2	interferon regulatory factor 2
12	91	cfa-miR-18b	PDE4D	phosphodiesterase 4D
13	91	cfa-miR-18b	LOC610321	UBAP1-MVB12-associated (UMA)-domain containing protein 1
14	91	cfa-miR-18b	SOCS5	suppressor of cytokine signaling 5
15	90	cfa-miR-18b	HIF1A	hypoxia inducible factor 1 subunit alpha
16	89	cfa-miR-18b	ADD3	adducin 3
17	89	cfa-miR-18b	ZNF367	zinc finger protein 367
18	89	cfa-miR-18b	MAP3K1	mitogen-activated protein kinase kinase kinase 1

19	89	cfa-miR-18b	ASXL2	ASXL transcriptional regulator 2
20	89	cfa-miR-18b	HSF5	heat shock transcription factor 5
21	89	cfa-miR-18b	KPNA6	karyopherin subunit alpha 6
22	89	cfa-miR-18b	SNAP23	synaptosome associated protein 23
23	89	cfa-miR-18b	GIGYF1	GRB10 interacting GYF protein 1
24	88	cfa-miR-18b	TMEM230	transmembrane protein 230
25	88	cfa-miR-18b	FBXL3	F-box and leucine rich repeat protein 3
26	88	cfa-miR-18b	PNISR	PNN interacting serine and arginine rich protein
27	88	cfa-miR-18b	GOLGA3	golgin A3
28	87	cfa-miR-18b	GLRB	glycine receptor beta
29	86	cfa-miR-18b	SORBS2	sorbin and SH3 domain containing 2
30	86	cfa-miR-18b	EPB41L1	erythrocyte membrane protein band 4.1 like 1
31	86	cfa-miR-18b	CCN2	cellular communication network factor 2
32	86	cfa-miR-18b	FARP1	FERM, ARH/RhoGEF and pleckstrin domain protein 1
33	85	cfa-miR-18b	NACC1	nucleus accumbens associated 1
34	85	cfa-miR-18b	KCNA1	potassium voltage-gated channel subfamily A member 1
35	85	cfa-miR-18b	GPR37	G protein-coupled receptor 37
36	85	cfa-miR-18b	ITGA6	integrin subunit alpha 6
37	84	cfa-miR-18b	IKZF3	IKAROS family zinc finger 3
38	83	cfa-miR-18b	CTDSPL	CTD small phosphatase like

39	83	cfa-miR-18b	CPA3	carboxypeptidase A3
40	83	cfa-miR-18b	SH3BP4	SH3 domain binding protein 4
41	82	cfa-miR-18b	HSD17B13	hydroxysteroid 17-beta dehydrogenase 13
42	82	cfa-miR-18b	LYPD8	LY6/PLAUR domain containing 8
43	81	cfa-miR-18b	FRS2	fibroblast growth factor receptor substrate 2
44	81	cfa-miR-18b	TMEM170B	transmembrane protein 170B
45	81	cfa-miR-18b	INSYN2	inhibitory synaptic factor 2A
**Target Rank**	**Target Score**	**miRNA Name**	**Gene Symbol**	**Gene Description**
1	99	cfa-miR-18a	ESR1	estrogen receptor 1
2	99	cfa-miR-18a	PATJ	PATJ, crumbs cell polarity complex component
3	98	cfa-miR-18a	RORA	RAR related orphan receptor A
4	97	cfa-miR-18a	MAP7D1	MAP7 domain containing 1
5	97	cfa-miR-18a	BBX	BBX, HMG-box containing
6	96	cfa-miR-18a	MNDA	myeloid cell nuclear differentiation antigen
7	95	cfa-miR-18a	CREBL2	cAMP responsive element binding protein like 2
8	94	cfa-miR-18a	ERI1	exoribonuclease 1
9	94	cfa-miR-18a	ESF1	ESF1 nucleolar pre-rRNA processing protein homolog
10	93	cfa-miR-18a	TRAPPC8	trafficking protein particle complex 8
11	93	cfa-miR-18a	IRF2	interferon regulatory factor 2
12	91	cfa-miR-18a	PDE4D	phosphodiesterase 4D

13	91	cfa-miR-18a	LOC610321	UBAP1-MVB12-associated (UMA)-domain containing protein 1
14	91	cfa-miR-18a	SOCS5	suppressor of cytokine signaling 5
15	90	cfa-miR-18a	HIF1A	hypoxia inducible factor 1 subunit alpha
16	89	cfa-miR-18a	ADD3	adducin 3
17	89	cfa-miR-18a	ZNF367	zinc finger protein 367
18	89	cfa-miR-18a	MAP3K1	mitogen-activated protein kinase kinase kinase 1
19	89	cfa-miR-18a	ASXL2	ASXL transcriptional regulator 2
20	89	cfa-miR-18a	HSF5	heat shock transcription factor 5
21	89	cfa-miR-18a	KPNA6	karyopherin subunit alpha 6
22	89	cfa-miR-18a	SNAP23	synaptosome associated protein 23
23	89	cfa-miR-18a	GIGYF1	GRB10 interacting GYF protein 1
24	88	cfa-miR-18a	TMEM230	transmembrane protein 230
25	88	cfa-miR-18a	FBXL3	F-box and leucine rich repeat protein 3
26	88	cfa-miR-18a	PNISR	PNN interacting serine and arginine rich protein
27	88	cfa-miR-18a	GOLGA3	golgin A3
28	87	cfa-miR-18a	GLRB	glycine receptor beta
29	86	cfa-miR-18a	SORBS2	sorbin and SH3 domain containing 2
30	86	cfa-miR-18a	EPB41L1	erythrocyte membrane protein band 4.1 like 1
31	86	cfa-miR-18a	CCN2	cellular communication network factor 2
32	86	cfa-miR-18a	FARP1	FERM, ARH/RhoGEF and pleckstrin domain protein 1

33	85	cfa-miR-18a	NACC1	nucleus accumbens associated 1
34	85	cfa-miR-18a	KCNA1	potassium voltage-gated channel subfamily A member 1
35	85	cfa-miR-18a	GPR37	G protein-coupled receptor 37
36	85	cfa-miR-18a	ITGA6	integrin subunit alpha 6
37	84	cfa-miR-18a	IKZF3	IKAROS family zinc finger 3
38	83	cfa-miR-18a	CTDSPL	CTD small phosphatase like
39	83	cfa-miR-18a	CPA3	carboxypeptidase A3
40	83	cfa-miR-18a	SH3BP4	SH3 domain binding protein 4
41	82	cfa-miR-18a	HSD17B13	hydroxysteroid 17-beta dehydrogenase 13
42	82	cfa-miR-18a	LYPD8	LY6/PLAUR domain containing 8
43	81	cfa-miR-18a	FRS2	fibroblast growth factor receptor substrate 2
44	81	cfa-miR-18a	TMEM170B	transmembrane protein 170B
45	81	cfa-miR-18a	INSYN2	inhibitory synaptic factor 2A
**Target Rank**	**Target Score**	**miRNA Name**	**Gene Symbol**	**Gene Description**
1	97	cfa-miR-21	FGF18	fibroblast growth factor 18
2	97	cfa-miR-21	PBRM1	polybromo 1
3	97	cfa-miR-21	ARHGAP24	Rho GTPase activating protein 24
4	96	cfa-miR-21	TGFBI	transforming growth factor beta induced
5	96	cfa-miR-21	ZNF367	zinc finger protein 367
6	96	cfa-miR-21	TTR	transthyretin

7	95	cfa-miR-21	SEC63	SEC63 homolog, protein translocation regulator
8	95	cfa-miR-21	SPART	spartin
9	94	cfa-miR-21	SPRY1	sprouty RTK signaling antagonist 1
10	94	cfa-miR-21	PLEKHA1	pleckstrin homology domain containing A1
11	94	cfa-miR-21	MAGOH	mago homolog, exon junction complex subunit
12	94	cfa-miR-21	CCDC32	coiled-coil domain containing 32
13	94	cfa-miR-21	YOD1	YOD1 deubiquitinase
14	94	cfa-miR-21	PELI1	pellino E3 ubiquitin protein ligase 1
15	94	cfa-miR-21	RALGPS2	Ral GEF with PH domain and SH3 binding motif 2
16	94	cfa-miR-21	IL12A	interleukin 12A
17	93	cfa-miR-21	GC	GC, vitamin D binding protein
18	93	cfa-miR-21	BCL7A	BCL7A, BAF complex component
19	93	cfa-miR-21	KLF3	Kruppel like factor 3
20	92	cfa-miR-21	ADGRG2	adhesion G protein-coupled receptor G2
21	92	cfa-miR-21	KRIT1	KRIT1, ankyrin repeat containing
22	92	cfa-miR-21	KDM7A	lysine demethylase 7A
23	91	cfa-miR-21	UIMC1	ubiquitin interaction motif containing 1
24	91	cfa-miR-21	ZNF287	zinc finger protein 287
25	91	cfa-miR-21	NTF3	neurotrophin 3
26	91	cfa-miR-21	ZNF804B	zinc finger protein 804B

27	91	cfa-miR-21	RMND5A	required for meiotic nuclear division 5 homolog A
28	90	cfa-miR-21	SOX5	SRY-box 5
29	90	cfa-miR-21	ST8SIA3	ST8 alpha-N-acetyl-neuraminide alpha-2,8-sialyltransferase 3
30	90	cfa-miR-21	JAG1	jagged 1
31	90	cfa-miR-21	WNK3	WNK lysine deficient protein kinase 3
32	90	cfa-miR-21	KLHL15	kelch like family member 15
33	90	cfa-miR-21	USPL1	ubiquitin specific peptidase like 1
34	89	cfa-miR-21	THAP5	THAP domain containing 5
35	89	cfa-miR-21	NFIA	nuclear factor I A
36	89	cfa-miR-21	RAD52	RAD52 homolog, DNA repair protein
37	89	cfa-miR-21	SEMA3D	semaphorin 3D
38	89	cfa-miR-21	GLIS2	GLIS family zinc finger 2
39	89	cfa-miR-21	GRAMD2B	GRAM domain containing 2B
40	88	cfa-miR-21	RRM2B	ribonucleotide reductase regulatory TP53 inducible subunit M2B
41	88	cfa-miR-21	ELF2	E74 like ETS transcription factor 2
42	88	cfa-miR-21	GATAD2B	GATA zinc finger domain containing 2B
43	87	cfa-miR-21	TRAPPC6B	trafficking protein particle complex 6B
44	87	cfa-miR-21	LTV1	LTV1 ribosome biogenesis factor
45	87	cfa-miR-21	CLDN8	claudin 8
46	86	cfa-miR-21	SKI	SKI proto-oncogene

47	86	cfa-miR-21	HNRNPU	heterogeneous nuclear ribonucleoprotein U
48	86	cfa-miR-21	CPT1C	carnitine palmitoyltransferase 1C
49	85	cfa-miR-21	TMEM170A	transmembrane protein 170A
50	85	cfa-miR-21	KRT20	keratin 20
51	85	cfa-miR-21	SC5D	sterol-C5-desaturase
52	85	cfa-miR-21	FNIP1	folliculin interacting protein 1
53	85	cfa-miR-21	C15H1orf185	chromosome 15 C1orf185 homolog
54	85	cfa-miR-21	PCSK6	proprotein convertase subtilisin/kexin type 6
55	85	cfa-miR-21	PPARA	peroxisome proliferator activated receptor alpha
56	84	cfa-miR-21	MBTPS2	membrane bound transcription factor peptidase, site 2
57	84	cfa-miR-21	PAN3	poly(A) specific ribonuclease subunit PAN3
58	84	cfa-miR-21	MAP3K1	mitogen-activated protein kinase kinase kinase 1
59	83	cfa-miR-21	C9H17orf75	chromosome 9 C17orf75 homolog
60	82	cfa-miR-21	PITX2	paired like homeodomain 2
61	82	cfa-miR-21	DNM1L	dynamin 1 like
62	82	cfa-miR-21	ARHGAP12	Rho GTPase activating protein 12
63	82	cfa-miR-21	KRT222	keratin 222
64	82	cfa-miR-21	OSR1	odd-skipped related transcription factor 1
65	82	cfa-miR-21	NFIB	nuclear factor I B
66	81	cfa-miR-21	UBE2D3	ubiquitin conjugating enzyme E2 D3

67	81	cfa-miR-21	MBLAC2	metallo-beta-lactamase domain containing 2
68	81	cfa-miR-21	SLC2A12	solute carrier family 2 member 12
69	81	cfa-miR-21	PDCD4	programmed cell death 4
**Target Rank**	**Target Score**	**miRNA Name**	**Gene Symbol**	**Gene Description**
1	99	cfa-miR-146b	IRAK1	interleukin 1 receptor associated kinase 1
2	98	cfa-miR-146b	NUMB	NUMB, endocytic adaptor protein
3	98	cfa-miR-146b	SEC23IP	SEC23 interacting protein
4	96	cfa-miR-146b	GHITM	growth hormone inducible transmembrane protein
5	94	cfa-miR-146b	NOVA1	NOVA alternative splicing regulator 1
6	92	cfa-miR-146b	MET	MET proto-oncogene, receptor tyrosine kinase
7	92	cfa-miR-146b	UNC5C	unc-5 netrin receptor C
8	92	cfa-miR-146b	TDRKH	tudor and KH domain containing
9	91	cfa-miR-146b	MMP13	matrix metallopeptidase 13
10	91	cfa-miR-146b	AHCYL2	adenosylhomocysteinase like 2
11	91	cfa-miR-146b	HNRNPD	heterogeneous nuclear ribonucleoprotein D
12	90	cfa-miR-146b	DCAF12	DDB1 and CUL4 associated factor 12
13	90	cfa-miR-146b	F2	coagulation factor II, thrombin
14	89	cfa-miR-146b	STRBP	spermatid perinuclear RNA binding protein
15	89	cfa-miR-146b	LOC102151519	zinc finger protein 300-like
16	88	cfa-miR-146b	C1H9orf40	chromosome 1 C9orf40 homolog

17	88	cfa-miR-146b	ARHGAP29	Rho GTPase activating protein 29
18	88	cfa-miR-146b	HORMAD1	HORMA domain containing 1
19	88	cfa-miR-146b	HIPK3	homeodomain interacting protein kinase 3
20	88	cfa-miR-146b	SLC11A2	solute carrier family 11 member 2
21	88	cfa-miR-146b	APPL1	adaptor protein, phosphotyrosine interacting with PH domain and leucine zipper 1
22	88	cfa-miR-146b	BCORL1	BCL6 corepressor like 1
23	87	cfa-miR-146b	DNAJB4	DnaJ heat shock protein family (Hsp40) member B4
24	87	cfa-miR-146b	MAD2L1	mitotic arrest deficient 2 like 1
25	87	cfa-miR-146b	CMC1	C-X9-C motif containing 1
26	87	cfa-miR-146b	NUDT12	nudix hydrolase 12
27	86	cfa-miR-146b	SHKBP1	SH3KBP1 binding protein 1
28	86	cfa-miR-146b	SPTLC3	serine palmitoyltransferase long chain base subunit 3
29	86	cfa-miR-146b	ESYT2	extended synaptotagmin 2
30	86	cfa-miR-146b	CD80	CD80 molecule
31	85	cfa-miR-146b	DEPTOR	DEP domain containing MTOR interacting protein
32	85	cfa-miR-146b	LOC483164	histone H2B type 2-F
33	85	cfa-miR-146b	KIF13B	kinesin family member 13B
34	84	cfa-miR-146b	GIT2	GIT ArfGAP 2
35	83	cfa-miR-146b	CCDC87	coiled-coil domain containing 87
36	83	cfa-miR-146b	PTPRA	protein tyrosine phosphatase, receptor type A

37	83	cfa-miR-146b	BAIAP2L1	BAI1 associated protein 2 like 1
38	83	cfa-miR-146b	KCNMB1	potassium calcium-activated channel subfamily M regulatory beta subunit 1
39	82	cfa-miR-146b	TET2	tet methylcytosine dioxygenase 2
40	82	cfa-miR-146b	ADO	2-aminoethanethiol dioxygenase
41	82	cfa-miR-146b	NEMP1	nuclear envelope integral membrane protein 1
42	81	cfa-miR-146b	ERBB4	erb-b2 receptor tyrosine kinase 4
43	81	cfa-miR-146b	CDS1	CDP-diacylglycerol synthase 1
44	81	cfa-miR-146b	THAP5	THAP domain containing 5
45	81	cfa-miR-146b	TRAF6	TNF receptor associated factor 6
46	81	cfa-miR-146b	VAPA	VAMP associated protein A
47	81	cfa-miR-146b	NPC2	NPC intracellular cholesterol transporter 2
